# Effects of important ecological measures on plant diseases and soil microbial communities in the Hulunber Grassland

**DOI:** 10.3389/fpls.2026.1868265

**Published:** 2026-10-16

**Authors:** Xin Yang, Xinyao Han, Xiaojun Rong, Kamran Malik, Xuan Tang, Rina Wu, Zhenming Zhang, Chunjie Li, Binhua Yu

**Affiliations:** 1 National Observation and Research Station of Grassland Agro-Ecosystem in Qingyang, Qingyang, Gansu, China; 2 College of Pastoral Agriculture Science and Technology, Lanzhou University, Lanzhou, China; 3 Engineering Research Center of Grassland Industry, Ministry of Education, Lanzhou University, Lanzhou, China; 4 Gansu Tech Innovation Center of Western China Grassland Industry, Lanzhou University, Lanzhou, China; 5 Grassland Research Center of National Forestry and Grassland Administration, Institute of Ecological Protection and Restoration, Chinese Academy of Forestry, Beijing, China; 6 Inner Mongolia Autonomous Region Plant Protection and Quarantine Center, Hohhot, China; 7 Hulunber Plant Protection and Quarantine Center, Hulunber, China; 8 Gansu Grassland Technical Extension Station, Lanzhou, China

**Keywords:** management measures, meadow grassland, microbial agent, plant diseases, soil microorganisms

## Abstract

**Introduction:**

Ecological management measures can influence plant diseases, forage production, soil nutrients, and microbial communities, but their integrated effects in natural meadow grasslands remain unclear. This study evaluated the effects of mowing twice per year, microbial agent application, and organic fertilizer application in the Hulunber natural grassland.

**Methods:**

Field plots were subjected to mowing twice per year (MT), microbial agent application (MA), and organic fertilizer application (OF), with untreated plots serving as the control (CK). Plant community and disease surveys, soil physicochemical analyses, and high-throughput sequencing were combined to assess treatment effects on plant community characteristics, foliar fungal diseases, soil nutrients, and soil microbial communities.

**Results:**

MA significantly increased plant community cover, whereas MT significantly reduced community height. MT increased fresh and hay forage yields by 89.26% and 85.62%, respectively, and OF also significantly increased forage yield. Leaf spot was the predominant plant disease, with peak incidence occurring in August. MA and MT significantly reduced community pathogen load, and MT produced the strongest reduction, decreasing community pathogen load by 56.60% relative to CK. At the functional-group level, MA and MT reduced pathogen loads in grasses and sedges, while all treatments reduced pathogen loads in legumes by 47.52%–65.33%. MA increased soil total nitrogen; MT increased total nitrogen, ammonium nitrogen, and soil organic carbon; and OF increased soil organic carbon. OF increased fungal alpha diversity, while bacterial diversity and community composition differed among treatments. MT increased the relative abundance of *Fusarium*, whereas MA, MT, and OF decreased the relative abundance of *Alternaria*.

**Discussion:**

Mowing twice per year and microbial agent application showed greater potential for reducing foliar fungal disease, whereas organic fertilizer mainly improved forage production, soil organic carbon, and fungal diversity. These findings provide an ecological basis for management strategies that balance disease reduction, forage production, and soil microbial conservation in meadow grasslands.

## 1 Introduction

Natural grasslands provide forage resources and support carbon sequestration, water regulation, soil conservation, and biodiversity maintenance (Wilcox et al., 2003; Scurlock and Hall, 2010; Bardgett and van der Putten, 2014). However, these ecosystems are sensitive to climate change and human use. Long-term or inappropriate use can alter plant community structure, soil nutrient cycling, and ecosystem resilience (Gang et al., 2014; Delavaux et al., 2020). Plant diseases are an important biotic stressor affecting grassland health. Pathogen infection can reduce photosynthetic performance and water and nutrient uptake, decrease forage yield and quality, and alter plant competition and community composition (Mitchell et al., 2002; Masumoto et al., 2018; Choudhary and Kumar, 2024). Grassland plant diseases have a broad distribution and substantial economic impact, with disease-associated yield losses in natural grasslands estimated at approximately 5% annually (Yan et al., 2023).

The Hulunber natural grassland supports species-rich plant communities and is an important forage-producing region. Regional surveys indicate that grassland disease occurrence is associated with vegetation cover, host composition, and grassland-use practices (Chen et al., 2018; Zhang, 2019). Foliar fungal diseases, particularly leaf spot diseases, are common during the growing season. Leaf spot diseases directly damage photosynthetic tissues and may reduce forage quality; their occurrence is related to host density, canopy structure, litter accumulation, near-surface microclimate, and primary inoculum sources (Fisher et al., 2012; Liu et al., 2016). Disease responses may also differ among plant species and functional groups because of variation in host status, phenology, and tissue traits. Consequently, studies based on a single host species cannot fully characterize disease dynamics in diverse natural grassland communities.

Chemical control, resistant cultivars, and agronomic practices are the principal approaches used to manage plant diseases (Strange and Scott, 2005; Wang et al., 2020). Their application in natural grasslands, however, is limited by the large area, high plant and pathogen diversity, spatial heterogeneity, and close linkage of these ecosystems with livestock production. Repeated fungicide use may be economically impractical and environmentally undesirable, whereas resistant cultivar development and replacement are primarily applicable to cultivated grasslands or individual dominant forage species (Nan, 2000; Ma et al., 2016). There is thus a need for management practices that are compatible with routine grassland use and can support both ecological function and forage production. Importantly, mowing, microbial agent application, and organic fertilizer application are grassland-use or ecological restoration practices. Their potential to reduce disease should be evaluated experimentally rather than assumed in advance.

Disease occurrence results from interactions among plant communities, soil conditions, microbial communities, and pathogens. Plant diversity, composition, and host density can influence pathogen transmission. High densities of susceptible dominant hosts may promote disease spread, whereas non-host or low-susceptibility species may interrupt transmission pathways through a dilution effect (Keesing et al., 2010; Ostfeld and Keesing, 2012; Rottstock et al., 2014). In turn, disease can alter community competition by selectively suppressing susceptible species (Alexander and Holt, 1998; Hudson et al., 2006). Soil microorganisms regulate organic matter decomposition and nutrient transformation and can affect plant health through resource competition, antibiosis, parasitism, and induced plant resistance (Joergensen and Emmerling, 2006; Mendes et al., 2013; Sánchez-Cañizares et al., 2017). Changes in soil nutrient availability may further influence disease indirectly through their effects on plant growth, tissue nutrient status, and rhizosphere microbial activity. Plant communities, soil nutrients, soil microbial communities, and disease-related variables should therefore be evaluated within an integrated plant–soil–microbe framework.

Microbial agents, mowing, and organic fertilizer application may affect grassland disease through distinct ecological pathways. Beneficial microorganisms in microbial agents may suppress pathogens directly or indirectly through niche competition, antimicrobial metabolite production, rhizosphere microbiome modification, and improved plant nutrient acquisition (Martínez-Medina et al., 2009; Su et al., 2023). For example, Bacillus species can fix nitrogen, mobilize phosphorus and potassium, and suppress some plant pathogens; they are therefore widely used in biofertilizers and biological control products (Cao et al., 2011; Lin et al., 2018). Nevertheless, their effects in the complex plant communities and heterogeneous soils of natural grasslands remain poorly understood.

Mowing may decrease foliar disease risk by removing infected tissues and pathogen-bearing residues, reducing primary inoculum, and altering canopy conditions (Fischer et al., 2012). It can also modify plant height, competitive dominance, diversity, and aboveground–belowground resource allocation (Hou et al., 1996; Doležal et al., 2019). However, mowing may also create wounds or, when applied at unsuitable times or frequencies, change host–pathogen interactions in ways that increase disease risk (Kobes et al., 2011; Li et al., 2021). Organic fertilizer can influence disease by supplying carbon and nutrients, improving soil structure, shifting microbial community composition, and promoting plant growth (Shi et al., 2006; Berthrong et al., 2013). Its effect is not necessarily suppressive: appropriately matured and applied organic fertilizer may promote disease-suppressive soil conditions, whereas inappropriate application may increase disease risk through nutrient imbalance or microbial shifts (Akanmu et al., 2021; Tang et al., 2023).

Although these practices may affect plant health, previous studies have mainly focused on croplands, cultivated grasslands, or individual host–pathogen systems. It remains unclear how microbial agents, mowing at different frequencies, and organic fertilizer simultaneously affect plant communities, soil nutrients, soil microbial communities, and disease occurrence in natural grasslands. In particular, few field studies have compared these measures under the same conditions and evaluated disease responses across population, functional-group, and community scales.

Accordingly, we established field plots in the Hulunber natural grassland subjected to mowing twice per year (MT), microbial agent application (MA), and organic fertilizer application (OF), with untreated plots as controls (CK). We hypothesized that: (1) MA would reduce community pathogen load by modifying soil microbial communities and improving plant growth; (2) mowing would affect foliar disease through removal of infected tissues and changes in plant communities and canopy conditions, with different effects between mowing frequencies; (3) OF would influence disease indirectly through changes in soil nutrients, plant communities, and soil microbial communities; and (4) greater plant diversity and soil microbial changes unfavorable to pathogen persistence would be associated with lower community pathogen load.

The objectives of this study were to: (1) determine the effects of MA, MT, and OF on plant community characteristics, forage productivity, and foliar fungal disease occurrence; (2) compare disease responses at population, plant functional-group, and community scales; and (3) examine treatment effects on soil nutrient properties and soil microbial community composition and diversity, and evaluate their associations with disease-related variables. The findings provide an ecological basis for plant disease management and sustainable use of natural grasslands.

## 2 Materials and methods

### 2.1 Experimental site

This study was conducted at the National Field Scientific Observatory of Hulunber Grassland Ecosystem, Chinese Academy of Agricultural Sciences (N49°19′- 21′, E119°55′- 58′). The experimental site is located in Xiertala Town, Hailar District, Hulunber City, Inner Mongolia, in the transition zone from the hilly areas of the Daxinganling Mountains to the Mongolian Plateau, at an altitude of 670–677 m. The site has a temperate semi-arid continental climate, with an average annual precipitation of 380–400 mm, an average annual temperature of -2°C-1°C, and a soil is characterized primarily by calcic chernozem. The grasslands in this area are utilized for grazing and mowing. The dominant species are *Leymus chinensis*, *Artemisia tanacetifolia*, *Thalictrum squarrosum*, and *Potentilla betonicifolia*. The accompanying species are *Bupleurum sibiricum*, *Vicia amoena*, *Pulsatilla turczaninovii*, and *Galium verum*.

#### 2.1.1 Site design and sample collection

The experimental site was established in 2024. A grassland area with flat terrain and homogeneous vegetation was selected within the study region. Experimental treatments were applied in late June, late July, and late August each year. To ensure comparable initial conditions among plots, plant community and disease surveys were conducted in all experimental plots before the formal implementation of treatments in late June 2024; no significant differences were detected among treatments. The experiment followed a randomized block design with four treatments: untreated control (CK), mowing twice per year (MT), microbial agent application (MA), and organic fertilizer application (OF). Each treatment included five replicate plots (5 m × 5 m), resulting in a total of 20 plots. A 1-m-wide buffer strip was established between adjacent plots to minimize interference among treatments caused by water and nutrient movement or pathogen dispersal.

Mowing was performed using a lawn mower at an approximate stubble height of 3 cm. The MT treatment was applied twice per year, in late June and late August; late June corresponds to the early growing season of grassland plants in the Hulunber region. Plant residues generated by mowing under the MT treatment were retained within the original plots. The MA and OF treatments were applied once in late June, late July, and late August each year, for a total of three applications per year.

The microbial agent used in this study was “Duowei Zengfeng,” supplied by Ningxia Zhongwei Taike Biotechnology Co., Ltd. It was a compound microbial agent composed of *Bacillus amyloliquefaciens*, *Beauveria bassiana*, and *Trichoderma harzianum*. The product registration certificate number was Microbial Fertilizer (2022) No. 11677, and the product complied with GB 20287–2006. The concentration of viable microorganisms was no less than 2.0 × 10^9^ CFU g^−1^. The microbial agent was applied at 4.0 kg ha^−1^ by foliar spraying.

The organic fertilizer was supplied by Inner Mongolia Yanglaoda Bio-organic Fertilizer Co., Ltd. It was a homogeneous granular product containing ≥30% organic matter and ≥4.0% total nutrients (N + P_2_O_5_ + K_2_O), including 1.91% total N, 1.19% P, and 1.08% K. Its moisture content was ≤30%, pH ranged from 5.5 to 8.5, and the seed germination index was ≥70%. The fertilizer was produced from fermented sheep manure through standardized aerobic fermentation and harmless treatment. It was applied at 125 kg ha^−1^ by surface broadcasting.

Plant disease surveys were conducted in mid-to-late June, July, and August of 2024 and 2025, 30 d after treatment application. In the CK, MA, MT, and OF treatments, three 0.5 m × 0.5 m quadrats were randomly selected from each replicate plot. With five replicate plots per treatment, a total of 60 quadrats were surveyed. Within each quadrat, the disease type and number of diseased individuals were recorded for each plant species. Disease severity was assessed according to the disease-rating criteria described by Nan and Li (2003).

After the disease survey, vegetation data were collected, including the cover, height, abundance, and frequency of all plant species. Subsequently, five 0.5 m × 0.5 m quadrats were selected from each plot using a five-point sampling method, resulting in a total of 100 quadrats (4 treatments × 5 plots × 5 quadrats). All aboveground plants within each quadrat were clipped at a height of 3 cm above the soil surface. Samples were placed in paper bags and weighed to determine fresh mass. They were then oven-dried at 65°C for 24 h to determine dry mass. The mean fresh and dry masses obtained from the five quadrats within each plot were used to represent the fresh and dry forage biomass of that plot. Before mowing in late June, fresh and dry forage biomass in the MT treatment was measured once more using the procedures described above. Based on the vegetation survey data of the sample plots, the species importance value (V), Margalef index (MI), Shannon’s diversity index (SI), Simpson’s diversity index (SDI) and Pielou’s species evenness index (PEI), Dominant species presence (D) were calculated.


$$\text{V}=\frac{\text{Pi}+\text{Cr}+\text{Hr}+\text{Dr}}{4}$$

$$\text{MI}=\frac{\text{SR}-1}{\ln\text{N}}$$

$$\text{SI}=-\sum_{i=1}^{n}V_{i}\;\text{ln}\;(V_{i})$$

$$\text{SDI}=1-\sum_{i=1}^{n}{V_{i}}^{2}$$

$$\text{PEI}=\frac{\text{SI}}{\ln\text{SR}}$$

$$\text{D}=\frac{\sum_{\text{i}=1}^{\text{n}}\text{Vi}}{\text{SR}}$$

Where Pi denotes the ratio of the number of individuals of the ith species to the total number of individuals in the community; Cr is the relative cover; Hr is the relative height; Dr is the relative frequency; SR (Species richness) is the number of species in the community.

After completing the plant disease survey, calculate the disease incidence (DI), disease severity (DS), and pathogen load of the community using the following formulas:


$$\text{DI}=\;\frac{\text{number}}{\text{total number}}\times 100\%$$

$$\text{DS}=\frac{\sum \text{grade}\times \text{number}}{\text{total number}\times \text{top grade}}\times 100$$

$$\text{Pathogen load}=\frac{\sum_{\text{i}=1}^{\text{n}}{\text{a}}_{\text{i}}{\text{V}}_{\text{i}}}{\sum_{i=1}^{\text{n}}{\text{a}}_{\text{i}}}$$

Where n is the number of plant species in the sample plot, ai is the cover of the i-th plant species, and Vi is the severity of disease of the i-th plant species.

#### 2.1.2 Identification of pathogens

Obligate parasitic pathogens cannot be isolated using conventional culture media; therefore, they were identified by microscopic examination. Tissues from the margins between diseased and healthy areas were selected based on typical symptoms and signs, such as powdery fungal growth and rust pustules. For obligate parasitic fungi, including powdery mildew fungi and rust fungi, transparent tape impressions and tissue sections were prepared as water mounts. Hyphae, haustoria, and diagnostic spores, such as urediniospores and teliospores, were observed under a light microscope to assess their morphology and arrangement.

Isolation, Purification, and Molecular Identification of Pathogenic Fungi. Potato dextrose agar (PDA) medium was prepared by boiling 200 g of diced potatoes in 1 L of distilled water for approximately 25 min. The extract was filtered, supplemented with 20 g glucose and 18 g agar, heated until completely dissolved, and brought to a final volume of 1 L with distilled water. The medium was autoclaved at 120 °C for 20 min. After cooling to approximately 70 °C, penicillin and streptomycin (approximately 100 mg each) were added. For fungal isolation, disinfected leaf tissues from the margins between diseased and healthy areas were cut into approximately 2-mm squares under aseptic conditions and placed on PDA medium. The plates were sealed and incubated at 23 °C, and fungal colonies were purified after 3–5 d. Genomic DNA was extracted from fungal mycelia using the OMEGA Fungal DNA Kit according to the manufacturer’s instructions. The fungal ITS region was amplified using the universal primers ITS1 and ITS4 in a 25-μL PCR reaction system. The PCR protocol consisted of an initial denaturation at 95 °C for 2.5 min, followed by 35 cycles of denaturation at 95 °C for 15 s, annealing at 54 °C for 30 s, and extension at 72 °C for 1 min, with a final extension at 72 °C for 10 min. Purified PCR products were sequenced, and the resulting sequences were compared with those in the NCBI database using BLAST for species-level identification.

#### 2.1.3 Soil sample collection and determination of physicochemical properties

Soil samples were collected using a five-point sampling method. Within each experimental plot, surface litter and the upper 1–2 cm of loose soil were removed before sampling. Soil cores with a diameter of 5 cm were collected to a depth of 10 cm at five sampling points. The soil was sieved in the field through 60- and 100-mesh sieves and then divided according to subsequent analyses. One subsample was sealed and stored in the dark at room temperature for routine soil physicochemical analyses. A second subsample was immediately placed in a portable low-temperature cooler and temporarily stored at −20 °C for soil microbial biomass determination. The remaining subsamples were transported to the laboratory under cold-chain conditions and immediately stored at −80 °C for high-throughput sequencing analysis of soil microbial communities.

Soil total nitrogen (TN) was determined using the Kjeldahl method. Air-dried soil samples passing through a 0.149-mm sieve were digested with concentrated H_2_SO_4_ and a catalyst mixture, followed by distillation using an automatic Kjeldahl analyzer and titration with standardized HCl.

Soil total phosphorus (TP) and total potassium (TK) were determined after HNO_3_–HF–HClO_4_ digestion. TP was measured using the molybdenum–antimony colorimetric method at 700 nm, and TK was determined by atomic absorption spectrophotometry.

Soil organic matter (SOM) was measured using the external-heating potassium dichromate oxidation method. Soil alkaline hydrolyzable nitrogen (AN) was determined using the alkaline hydrolysis diffusion method. Soil available phosphorus (AP) was determined by sodium bicarbonate extraction followed by molybdenum–antimony colorimetry at 700 nm. Soil available potassium (AK) was measured by neutral ammonium acetate extraction and atomic absorption spectrophotometry. Soil pH was determined potentiometrically using a calibrated pH meter. Reagent blanks were included in all analyses for quality control.

#### 2.1.4 DNA extraction and high-throughput sequencing of soil microorganisms

Total genomic DNA was extracted from soil samples using the TGuide S96 Magnetic Soil DNA Kit (Tiangen Biotechnology (Beijing) Co. Ltd.) The quality and quantity of extracted DNA was checked by electrophoresis on a 1.8% agarose gel, and the concentration and purity of the DNA were determined using a NanoDrop 2000 UV-visible spectrophotometer (Thermo Scientific, Wilmington, USA). The highly variable region V1-V9 of the bacterial 16S rRNA gene was amplified using primers (27F: AGRGTTTGATYNTGGCTCAG; 1492R: TASGGHTACCTTGTTASGACTT). Primers (ITS-1F: GGAAGTAAAAGTCGTAACAAGG; ITS-2R: GCTGCGTTCTTCATCGATGC) amplified the internal transcribed spacer 1 region of fungal ribosomal DNA. The amplicons were quantified, and then the normalized equimolar concentrations of the amplicons were combined and sequenced on the PacBio Sequel II platform (Beijing Biomarker Technology Co., Ltd., Beijing, China).Eligible sequences with a similarity threshold of more than 97% were assigned to an Operational Taxonomic Unit (OTU) using USEARCH (version 10.0) Classification annotation of OTUs/ASVs was performed based on the Naive Bayes classifier in QIIME2 using the SILVA database (version 138.1), with a confidence threshold of 70%.

### 2.2 Data analysis

All experimental data were initially organized in Microsoft Excel 2021. Before formal statistical analysis, all data were tested for normality using the Shapiro-Wilk test and for homogeneity of variance using Levene’s test. When the assumptions were not met, data were transformed using arcsine-square root or log transformation to satisfy the requirements of ANOVA. Statistical analyses were performed using SPSS 26.0, and data visualization was conducted using R 4.5.2 and GraphPad Prism 9.5.0.

For plant community characteristics, plant disease severity, soil physicochemical nutrients, and the relative abundance of soil pathogenic fungi, one-way ANOVA followed by Duncan’s multiple comparison test was used to compare differences among treatments. For comparisons between two independent groups, independent-samples t-tests were used, with the significance level set at P< 0.05. General linear models were used to fit the relationships between plant community disease occurrence and plant community diversity. For alpha diversity indices (e.g., Simpson and Shannon), the same assumption checks were applied, and one-way ANOVA followed by Duncan’s test was used to compare treatment differences.

For high-throughput sequencing data, quality-filtered effective sequences were clustered into operational taxonomic units (OTUs) using USEARCH 10.0 at a 97% similarity threshold. Taxonomic annotation of OTUs/ASVs was performed using the Naive Bayes classifier in QIIME2 with the SILVA database (version 138.1) at a confidence threshold of 70%. Alpha-diversity analysis was performed using QIIME2. For soil bacterial and fungal communities, the alpha diversity indices calculated included observed features (OTUs/ASVs), ACE, Chao1, Simpson, Shannon, and coverage. For plant communities, species richness, Margalef richness index, Shannon diversity index, Simpson diversity index, Pielou’s evenness index, and dominant species presence index were calculated from the vegetation survey data. Non-metric multidimensional scaling (NMDS) and non-parametric multivariate analysis of variance (ADONIS) based on the Bray-Curtis distance were used to test for differences in microbial community composition among treatments.

## 3 Results

### 3.1 Effects of different treatments on plant communities

Compared with CK, MA treatment increased plant community cover significantly by 16% (*P<* 0.05). MT and OF treatments also increased community cover in trend by 4% and 9% respectively, although not significantly. Whereas, MT treatment significantly decreased plant community height by 19% (*P<* 0.05), and both MT and OF treatments significantly (*P<* 0.05) increased fresh and hay production, with fresh grass production increasing by 89% and 17% and hay production increasing by 86% and 13%, respectively (**Figure 1**).

**Figure 1:**
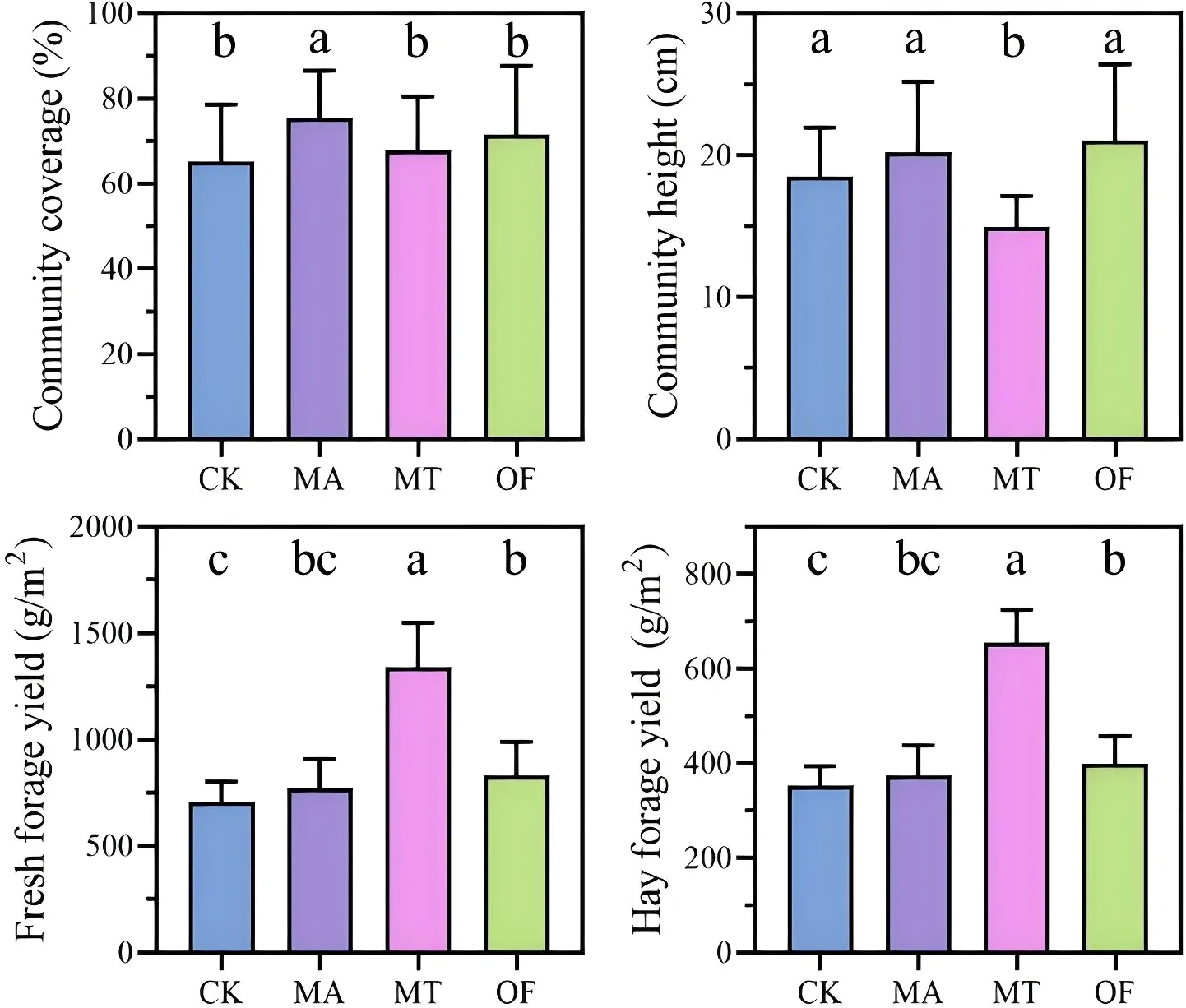
Effects of different treatments on community coverage, height and forage yield. Different lowercase letters indicate significant differences at P < 0.05; CK, control check; MA, microbial agent; MT, mowing; OF, organic fertilizer.

The MA treatment significantly (*P<* 0.05) decreased the Pielou and Simpson indices of the plant community by 3.2% and 2.1%, respectively, compared with the control; the remaining treatments were not significant in terms of species richness, Margalef index and Shannon’s diversity index. However, the MA treatment and MT treatment significantly increased (*P<* 0.05) the dominant species presence index by 6.3% and 6.6%, respectively (**Figure 2**).

**Figure 2:**
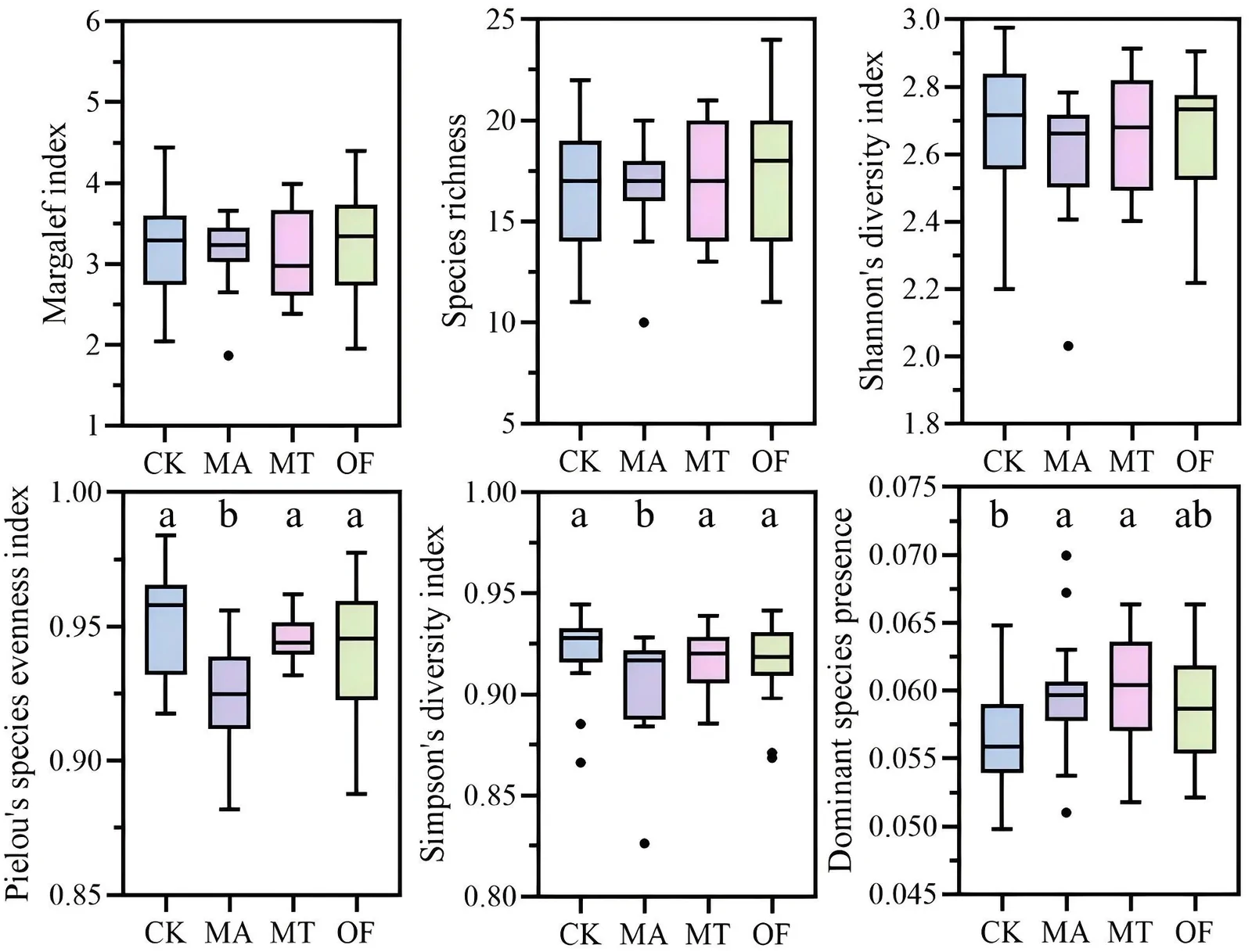
Effects of different treatments on plant community alpha diversity indices. Different lowercase letters indicate significant differences at P < 0.05; CK, control check; MA, microbial agent; MT, mowing; OF, organic fertilizer.

### 3.2 Effects of different treatments on plant diseases

#### 3.2.1 Host plants and diseases

A total of 23 plant families, 54 plant genera, and 67 plant species were identified in the study area. Of these, 15 families, 39 genera, and 48 species were found to be diseased, accounting for 71.6% of the total plant species identified. Leaf spot disease was observed in 47 species, rust disease in four species, and powdery mildew was prevalent in five species. A visual presentation of common diseases including leaf spots on *Thalictrum squarrosum*, *Leymus chinensis* and *Potentilla betonicifolia*; rusts on *Adenophora gmelinii* among others, is shown in **Figure 3A**.

**Figure 3:**
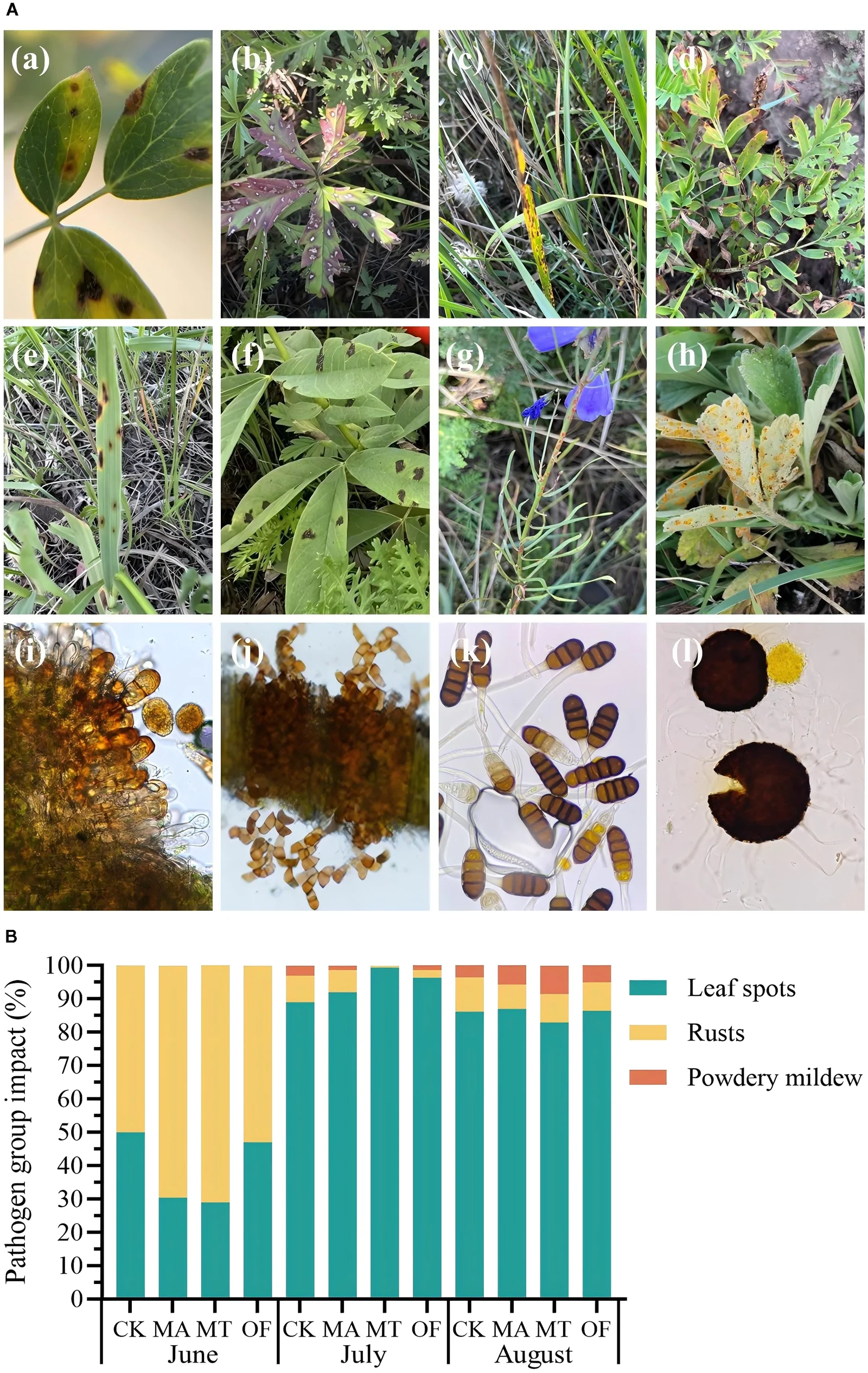
**(A)** Common diseased plants. **(a)** Leaf spots on Thalictrum squarrosum; **(b)** Leaf spots on Potentilla belenlicifolia; **(c)** Leaf spots on Leymus chinensis; **(d)** Leaf spots on Polenilla bifurca; **(e)** Leaf spots on Bromus inermis; **(f)** Leaf spots on Thermopsis lanceolata; **(g)** Rusts on Adenophora gmelinii; **(h)** Rusts on Potentilla acaulis; **(i)** Rusts caused by Puccinia sp. on Thalictrum squarrosum; **(j)** Rusts caused by Puccinia sp. on Bromus inermis; **(k)** Rusts caused by Phragmidium potenlillae on Potentilla tanacetifolia; **(l)** Powdery mildew caused by **(E)** pisi on Astragalus adsurgens. **(B)** The impact of the pathogen group on leaf spot disease, rust disease, and powdery mildew was examined under different treatments in June, July, and August. CK, control check; MA, microbial agent; MT, mowing; OF, organic fertilizer.

Throughout the growing season, disease incidence exhibited distinct temporal dynamics, with June marking the peak onset of rust disease. Under MA and MT treatments, pathogen infection rates reached 69% and 71%, respectively. However, after July, the MT and OF treatments significantly reduced rust disease infection levels, with infection rates decreasing by 92% and 72%, respectively, compared to the control group. The occurrence of leaf spot disease was concentrated in July and August, during which the pathogen infection rate remained above 85%, making it the predominant disease during this period. Meanwhile, powdery mildew initiated to appear from the mid-growing season and continued to spread, reaching its peak infection level by the end of the growing season. Particularly in August, the pathogen infection rate for powdery mildew exceeded 8% under MT treatment (**Figure 3B**).

#### 3.2.2 Plant disease incidence, disease severity, and pathogen load

For different plant functional groups and plant communities as a whole, MA treatment significantly reduced pathogen loads in Gramineae, Asteraceae, and plant communities. MT treatment significantly reduced pathogen loads in Gramineae, Asteraceae, Fabaceae, and plant communities, while OF treatment had no significant effect on pathogen loads (**Figure 4**).

**Figure 4:**
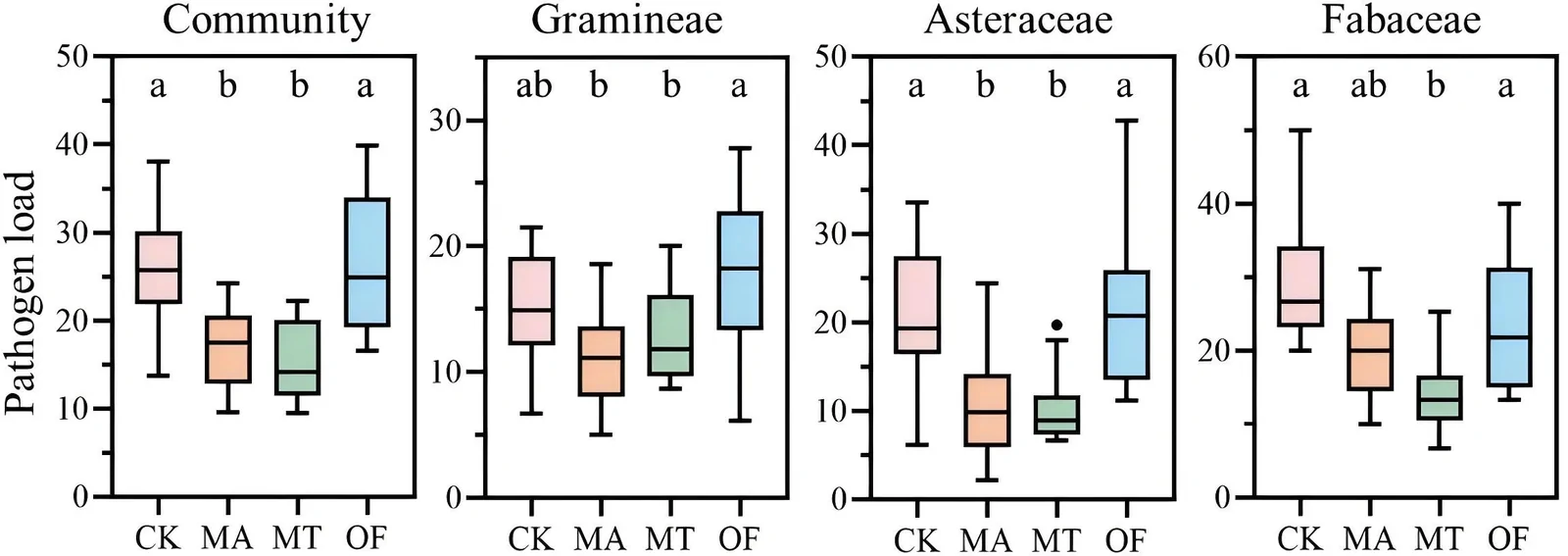
Effects of different treatments on pathogen loads in the plant community and plant functional groups. Different lowercase letters indicate significant differences at P < 0.05; CK, control check; MA, microbial agent; MT, mowing; OF, organic fertilizer.

Data analysis indicates that August represents the peak period for plant disease outbreaks in this region, with 56 distinct diseases detected across 48 plant species. Among these, facultative parasitic diseases occurred on 47 plant species, while obligate parasitic diseases were observed on only 9 plant species (**Table 1**). Different ecological management practices exert a greater influence on the incidence frequency and severity of facultative parasitic diseases. The survey found that *Leymus chinensis*, *Thermopsis lanceolata* and *Thalictrum squarrosum* were the most common species with the highest incidence and severity of disease in the experimental plots in August., with disease severity in the control plots being 31.04, 32.56, and 30.54, respectively. Under MA and MT treatments, the incidence rates and severity of leaf spot disease in *Leymus chinensis* and *Thermopsis lanceolata* both decreased. However, under OF treatment, the incidence rate and severity of leaf spot disease in *Thalictrum squarrosum* increased. The incidence and severity of leaf spot disease were reduced for dominant species *Pulsatilla turczaninovii* and *Artemisia tanacetifolia* under MA and MT treatments (**Table 1**). Under MA, MT, and OF treatments, the incidence and severity of disease decreased for *Adenophora gmelinii* rust (**Table 2**). Different treatments had significant effects on plant diseases at the species level. These effects were evident in the incidence and disease index of 15 plant species. The strongest effect was on the incidence of leaf spot disease in *Galiun verum* (F = 19.99, *P* < 0.001).

**Table 1:** Disease incidence (DI) and severity (DS) of host plant species under different treatments in August. (Facultative parasitic).

Host plant species	Disease type	Disease metric	CK	MA	MT	OF	F	*P*
*Allium bidentatum*	Leaf spots	DI(%)	100.00	92.86 ± 18.90	90.00 ± 21.08	97.78 ± 6.67	1.12	>0.05
		DS	40.00 ± 17.19	25.81 ± 15.73	26.15 ± 12.45	34.86 ± 14.28	2.10	>0.05
*Allium senescens*	Leaf spots	DI(%)	97.62 ± 6.30	50.00 ± 13.61	58.26 ± 24.71	86.11 ± 22.15	8.59	<0.01
		DS	21.43 ± 2.62	10.00 ± 2.72	11.65 ± 4.94	18.56 ± 6.26	8.09	<0.01
*Allium tenuissimum*	Leaf spots	DI(%)	**65.48 ± 25.65**	**36.41 ± 17.53**	**29.66 ± 13.38**	**77.82 ± 24.63**	10.68	<0.001
		DS	**13.10 ± 5.13**	**7.28 ± 3.51**	**5.93 ± 2.68**	**17.13 ± 5.03**	13.38	<0.001
*Artemisia argyi*	Leaf spots	DI(%)	38.89 ± 7.86	0*	40.00*	100.00	—	—
		DS	7.78 ± 1.57	0*	8.00*	20.00*	—	—
*Artemisia dracunculus*	Leaf spots	DI(%)	95.00 ± 12.25	80.65 ± 22.26	66.22 ± 20.31	100.00	3.92	<0.05
		DS	30.60 ± 8.52	20.95 ± 8.24	13.24 ± 4.06	26.79 ± 8.51	5.20	<0.05
*Artemisia tanacetifolia*	Leaf spots	DI(%)	**79.63 ± 21.96**	**39.76 ± 21.19**	**42.57 ± 14.49**	**83.89 ± 19.34**	15.59	<0.001
		DS	**19.43 ± 5.57**	**8.47 ± 4.88**	**8.93 ± 2.93**	**26.75 ± 12.53**	16.34	<0.001
*Astragalus adsurgens*	Leaf spots	DI(%)	NA	NA	NA	41.67 ± 11.79	—	—
		DS	NA	NA	NA	8.33 ± 2.36	—	—
*Astragalus melilotoides*	Leaf spots	DI(%)	100.00	100.000	NA	NA	—	—
		DS	20.00*	20.00*	NA	NA	—	—
*Bromus inermis*	Leaf spots	DI(%)	100.00	79.48 ± 17.70	78.09 ± 14.68	85.00 ± 33.54	1.35	>0.05
		DS	24.36 ± 2.73	19.23 ± 9.73	17.78 ± 6.41	24.67 ± 12.55	0.85	>0.05
*Bupleurum scorzonerifolium*	Leaf spots	DI(%)	**77.71 ± 31.70**	**69.82 ± 17.00**	**32.47 ± 25.35**	**79.35 ± 21.19**	9.02	<0.001
		DS	22.04 ± 12.48	15.27 ± 3.85	6.59 ± 5.12	20.14 ± 9.89	7.09	<0.01
*Carex duriuscula*	Leaf spots	DI(%)	71.70 ± 16.65	66.74 ± 16.70	67.82 ± 16.47	62.50 ± 19.00	0.68	>0.05
		DS	19.73 ± 5.50	17.09 ± 4.45	17.37 ± 4.41	16.14 ± 5.30	1.38	>0.05
*Carex pediformis*	Leaf spots	DI(%)	NA	83.33 ± 23.57	NA	83.33 ± 33.33	0.01	>0.05
		DS	NA	16.67 ± 4.71	NA	24.00 ± 11.37	0.70	>0.05
*Cerastium arvense*	Leaf spots	DI(%)	NA	100.00	NA	30.00*	—	—
		DS	NA	30.00*	NA	14.55*	—	—
*Cleistogenes squarrosa*	Leaf spots	DI(%)	61.48 ± 21.88	42.22 ± 10.49	44.78 ± 16.02	67.46 ± 20.63	4.82	<0.01
		DS	12.42 ± 4.31	8.45 ± 2.10	8.96 ± 3.20	15.30 ± 5.92	6.13	<0.01
*Dianthus chinensis*	Leaf spots	DI(%)	NA	100.00	NA	100.00	—	—
		DS	NA	20.00*	NA	20.00*	—	—
*Erigeron acer*	Leaf spots	DI(%)	100.00	50.00*	80 ± 28.28	100.00	3.81	>0.05
		DS	32.35 ± 12.86	10.00*	40 ± 28.28	20.00*	0.74	>0.05
*Galiun verum*	Leaf spots	DI(%)	**79.26 ± 21.75**	**85.09 ± 18.28**	**33.63 ± 14.89**	**87.62 ± 18.22**	19.99	<0.001
		DS	**19.42 ± 9.62**	**18.51 ± 5.28**	**6.73 ± 2.98**	**29.99 ± 16.14**	9.94	<0.001
*Gueldenstaedtia multiflora*	Leaf spots	DI(%)	100.00	NA	NA	NA	—	—
		DS	20.00*	NA	NA	NA	—	—
*Klasea centauroides*	Leaf spots	DI(%)	50.00*	NA	NA	100.00	—	—
		DS	10.00*	NA	NA	20.00*	—	—
*Koeleria cristata*	Leaf spots	DI(%)	40.00 ± 9.13	48.61 ± 30.47	100.00	57.03 ± 34.60	1.35	>0.05
		DS	8.00 ± 1.83	11.19 ± 6.78	20.00*	13.65 ± 9.91	1.17	>0.05
*Koenigia divaricata*	Leaf spots	DI(%)	100.00	100.00	96.00 ± 8.94	100.00	0.92	>0.05
		DS	28.31 ± 6.22	22.44 ± 2.50	24.53 ± 9.46	36.67 ± 5.77	3.30	<0.05
*Lactuca sibirica*	Leaf spots	DI(%)	100.00	100.00	NA	100.00	—	—
		DS	20.00*	20.00*	NA	33.75 ± 8.84	—	—
*Leymus chinensis*	Leaf spots	DI(%)	96.10 ± 1.11	98.36 ± 3.17	91.42 ± 15.48	96.67 ± 1.15	0.57	>0.05
		DS	**31.04 ± 10.38**	**22.74 ± 2.31**	**22.32 ± 8.88**	**31.14 ± 9.88**	2.91	<0.05
*Linaria vulgaris subsp. chinensis*	Leaf spots	DI(%)	100.00	41.67 ± 11.79	33.33*	100.00	—	—
		DS	35.00*	8.33 ± 2.36	6.67*	20.00*	—	—
*lris ventricosa*	Leaf spots	DI(%)	100.00	100.00	100.00	100.00	—	—
		DS	20.00*	20.00*	20.00*	30.00 ± 14.14	0.20	>0.05
*Oxytropis miyriophylla*	Leaf spots	DI(%)	NA	NA	100.00	NA	—	—
		DS	NA	NA	30.00*	NA	—	—
*Plantago depressa*	Leaf spots	DI(%)	75.00 ± 35.36	NA	NA	NA	—	—
		DS	20.00*	NA	NA	NA	—	—
*Poa annua*	Leaf spots	DI(%)	92.31*	NA	NA	51.52 ± 21.43	2.42	>0.05
		DS	18.46*	NA	NA	10.30 ± 4.29	2.42	>0.05
*Polenlilla bifurca*	Leaf spots	DI(%)	95.56 ± 13.33	72.75 ± 20.44	73.33 ± 26.82	86.26 ± 15.79	1.93	>0.05
		DS	29.11 ± 10.11	17.75 ± 9.12	18.83 ± 12.65	22.14 ± 7.59	1.51	>0.05
*Potentilla acaulis*	Leaf spots	DI(%)	56.25 ± 26.99	NA	23.30 ± 13.70	47.18 ± 31.10	2.88	>0.05
		DS	13.33 ± 8.02	NA	5.20 ± 3.51	9.78 ± 6.03	2.84	>0.05
*Potentilla betonicifolia*	Leaf spots	DI(%)	56.41*	24.74 ± 8.73	59.52 ± 1.57	70.55 ± 26.69	3.93	<0.05
		DS	15.9*	6.33 ± 4.22	29.11 ± 3.93	26.82 ± 15.69	2.85	>0.05
*Potentilla tanacetifolia*	Leaf spots	DI(%)	59.17 ± 32.91	55.48 ± 41.20	44.92 ± 38.21	73.33 ± 30.77	1.43	>0.05
		DS	14.97 ± 8.87	11.67 ± 7.99	8.98 ± 7.64	21.33 ± 19.75	1.73	>0.05
*Pulsatilla turczaninovii*	Leaf spots	DI(%)	84.80 ± 21.70	55.56 ± 34.43	52.81 ± 28.45	87.80 ± 16.44	5.52	<0.05
		DS	26.80 ± 15.44	12.61 ± 9.31	12.56 ± 8.93	25.96 ± 10.25	4.47	<0.05
*Sanguisorba officinalis*	Leaf spots	DI(%)	NA	NA	100.00	NA	—	—
		DS	NA	NA	60.00*	NA	—	—
*Saposhnikovia divaricata*	Leaf spots	DI(%)	100.00	100.00	NA	100.00	—	—
		DS	20.00*	60.00*	NA	20.00*	—	—
*Saussurea amara*	Leaf spots	DI(%)	NA	NA	100.00	76.67 ± 25.17	0.65	>0.05
		DS	NA	NA	20.00*	30.33 ± 25.70	0.12	>0.05
*Schizonepeta multifida*	Leaf spots	DI(%)	74.07 ± 44.91	0.00*	53.57 ± 5.05	NA	3.30	>0.05
		DS	28.70 ± 25.34	0.00*	10.71 ± 1.01	NA	1.63	>0.05
*Scorzonera ausriaca*	Leaf spots	DI(%)	88.89 ± 19.25	100.00	0.00*	80.00 ± 40.00	10.21	<0.01
		DS	20.00*	20.00*	0.00*	26.00 ± 17.44	3.73	<0.05
*Sibbaldia adpressa*	Leaf spots	DI(%)	75.00 ± 35.36	50.00*	87.78 ± 10.72	NA	1.10	>0.05
		DS	15.00 ± 7.07	10.00*	20.89 ± 5.39	NA	1.42	>0.05
*Sonchus oleraceus*	Leaf spots	DI(%)	66.67 ± 38.49	75.00*	33.33*	73.28 ± 20.90	0.47	>0.05
		DS	13.33 ± 7.70	15.00*	6.67*	20.32 ± 8.65	0.95	>0.05
*Stilpnolepis centiflora*	Leaf spots	DI(%)	83.33 ± 27.89	100.00	47.22 ± 20.97	100.00	3.08	>0.05
		DS	17.78 ± 6.89	26.67 ± 11.55	9.44 ± 4.19	20.00*	2.50	>0.05
*Stipa Baicalensis*	Leaf spots	DI(%)	75.00 ± 35.36	100.00	90.00 ± 22.36	90.00 ± 22.36	0.41	>0.05
		DS	15.00 ± 7.07	24.00 ± 5.66	20.00 ± 7.07	22.00 ± 10.95	0.43	>0.05
*Taraxacum mongolicum*	Leaf spots	DI(%)	NA	50.00	58.33 ± 28.87	NA	0.07	>0.05
		DS	NA	10.00	11.67 ± 5.77	NA	0.07	>0.05
*Tephroseris kirlowii*	Leaf spots	DI(%)	56.40 ± 30.65	34.58 ± 18.13	76.00 ± 32.86	67.86 ± 29.88	1.95	>0.05
		DS	14.26 ± 8.98	7.33 ± 4.45	26.00 ± 15.23	18.75 ± 11.29	2.97	>0.05
*Thalictrum squarrosum*	Leaf spots	DI(%)	**100.00**	**98.70 ± 4.31**	**73.15 ± 22.85**	**100.00**	12.43	<0.001
		DS	**32.56 ± 4.58**	**26.08 ± 4.58**	**16.56 ± 5.72**	**35.08 ± 11.37**	11.93	<0.001
*Thermopsis lanceolata*	Leaf spots	DI(%)	100.00	82.78 ± 23.51	64.70 ± 26.29	86.46 ± 14.73	5.82	<0.05
		DS	30.54 ± 10.18	23.51 ± 10.86	15.68 ± 7.35	30.63 ± 22.87	2.85	>0.05
*Vicia amoena*	Leaf spots	DI(%)	100.00	87.22 ± 13.69	65.79 ± 23.64	96.30 ± 9.07	4.70	<0.05
		DS	23.00 ± 2.65	18.69 ± 5.22	13.99 ± 4.26	28.15 ± 9.42	4.49	<0.05

Values represent mean ± standard error; NA (not available) indicates that this plant was not found in this treatment; * indicates that this plant could not be calculated as a standard error (SE) in this treatment due to the small number of replicates. — indicates that the number of replicates for this species is too small to perform a one-way analysis of variance.

Bold values indicate a significant overall treatment effect according to one-way analysis of variance (P < 0.05).

**Table 2:** Disease incidence (DI) and severity (DS) of host plant species under different treatments in August. (Obligate parasitic).

Host plant species	Disease type	Disease metric	CK	MA	MT	OF	F	*P*
*Artemisia tanacetifolia*	Powdery mildew	DI(%)	NA	NA	27.38 ± 31.99	NA	—	—
		DS	NA	NA	9.92 ± 9.54	NA	—	—
*Astragalus adsurgens*	Powdery mildew	DI(%)	NA	NA	NA	66.67	—	—
		DS	NA	NA	NA	22.22	—	—
*Galiun verum*	Rusts	DI(%)	67.38 ± 32.26	61.76 ± 23.45	55.32 ± 26.92	78.44 ± 27.50	1.577	>0.05
		DS	**21.87 ± 17.16**	**17.38 ± 9.18**	**13.83 ± 6.73**	**30.11 ± 17.82**	3.32	<0.05
*Leymus chinensis*	Rusts	DI(%)	39.30 ± 17.15	31.26 ± 12.41	26.44 ± 9.45	50.68 ± 30.31	1.991	>0.05
		DS	9.91 ± 4.14	7.81 ± 3.10	6.61 ± 2.36	12.67 ± 7.58	2.026	>0.05
*Polenlilla bifurca*	Powdery mildew	DI(%)	NA	NA	100.00	15.38*	—	—
		DS	NA	NA	66.67*	10.26*	—	—
*Potentilla acaulis*	Rusts	DI(%)	NA	NA	32.60 ± 19.17	NA	—	—
		DS	NA	NA	8.15 ± 4.79	NA	—	—
*Tephroseris kirlowii*	Powdery mildew	DI(%)	12.50*	NA	NA	32.14 ± 25.25	0.403	>0.05
		DS	12.50*	NA	NA	10.71 ± 8.42	0.03	>0.05
*Thalictrum squarrosum*	Powdery mildew	DI(%)	95.71 ± 8.08	88.44 ± 22.81	88.73 ± 19.71	98.62 ± 4.36	0.97	>0.05
		DS	53.06 ± 15.10	38.44 ± 14.35	40.71 ± 12.70	50.64 ± 10.88	2.779	>0.05
*Adenophora gmelinii*	Rusts	DI(%)	100.00	91.43 ± 22.68	100.00	91.67 ± 15.43	0.43	>0.05
		DS	61.67 ± 55.08	31.13 ± 14.46	43.75 ± 37.50	49.31 ± 24.17	0.891	>0.05

Values represent mean ± standard error; NA (not available) indicates that this plant was not found in this treatment; * indicates that this plant could not be calculated as a standard error (SE) in this treatment due to the small number of replicates. — indicates that the number of replicates for this species is too small to perform a one-way analysis of variance.

Bold values indicate a significant overall treatment effect according to one-way analysis of variance (P < 0.05).

### 3.3 Effects of different treatments on soil microorganisms in grasslands

#### 3.3.1 Effects on grassland soil nutrients

As shown in **Table 3**, the microbial agent, mowing, and fertilization treatments significantly affected the soil’s nutrient content. The MA treatment increased the soil’s total nitrogen content by 46% (*P<* 0.05) compared to the CK treatment. The MT treatment increased the soil’s total nitrogen, ammonium nitrogen, and organic carbon content by 63.6%, 34.8%, and 31.3%, respectively. The OF treatment increased the organic carbon content by 21.5% (*P<* 0.05). Different ecological management practices have increased soil nutrients to varying degrees.

**Table 3:** Physical and chemical characteristics of soil under different grassland management regime.

Soil nutrients	CK	MA	MT	OF
Total nitrogen (mg/g)	1.62 ± 0.09b	2.37 ± 0.26a	2.65 ± 0.24a	1.67 ± 0.68b
Total phosphorus (mg/g)	0.47 ± 0.07a	0.49 ± 0.05a	0.4 ± 0.06a	0.42 ± 0.07a
Ammonium nitrogen (mg/kg)	1.35 ± 0.31b	1.43 ± 0.18b	1.82 ± 0.09a	1.4 ± 0.22b
Nitrate nitrogen (mg/g)	0.09 ± 0.01a	0.08 ± 0.01a	0.07 ± 0.02a	0.08 ± 0.02a
Available potassium (mg/g)	0.57 ± 0.09a	0.66 ± 0.18a	0.7 ± 0.18a	0.71 ± 0.18a
Organic carbon (mg/g)	22.97 ± 1.72c	23.95 ± 2.29bc	30.16 ± 1a	27.92 ± 5.35ab
pH	6.87 ± 0.18a	6.76 ± 0.33a	6.77 ± 0.2a	6.86 ± 0.16a

Values represent mean ± standard error; Different lowercase letters indicate significant differences at *P* < 0.05; CK, control check; MA, microbial agent; MT, mowing; OF, organic fertilizer.

#### 3.3.2 Effects on the diversity of microbial communities in grassland soils

The fungal ITS region was amplified using DNA from soil microorganisms and the highly variable region V1-V9 of the bacterial 16S rRNA gene was amplified. The fungal community got 2821 OTUs, and the number of OTUs specific to the CK, MA, MT and OF treatments were 154, 175, 190 and 241, respectively, for a total of 914 OTUs, with the OF treatment having the most fungal OTUs specific to it (**Figure 5a**). The bacterial community obtained 24,082 OTUs, the number of OTUs specific to CK, MA, MT and OF treatments were 1106, 1178, 1763 and 1348, respectively, and the total number of OTUs was 5238, of which the MT treatment had the highest number of bacterial OTUs specific to it (**Figure 5b**).

**Figure 5:**
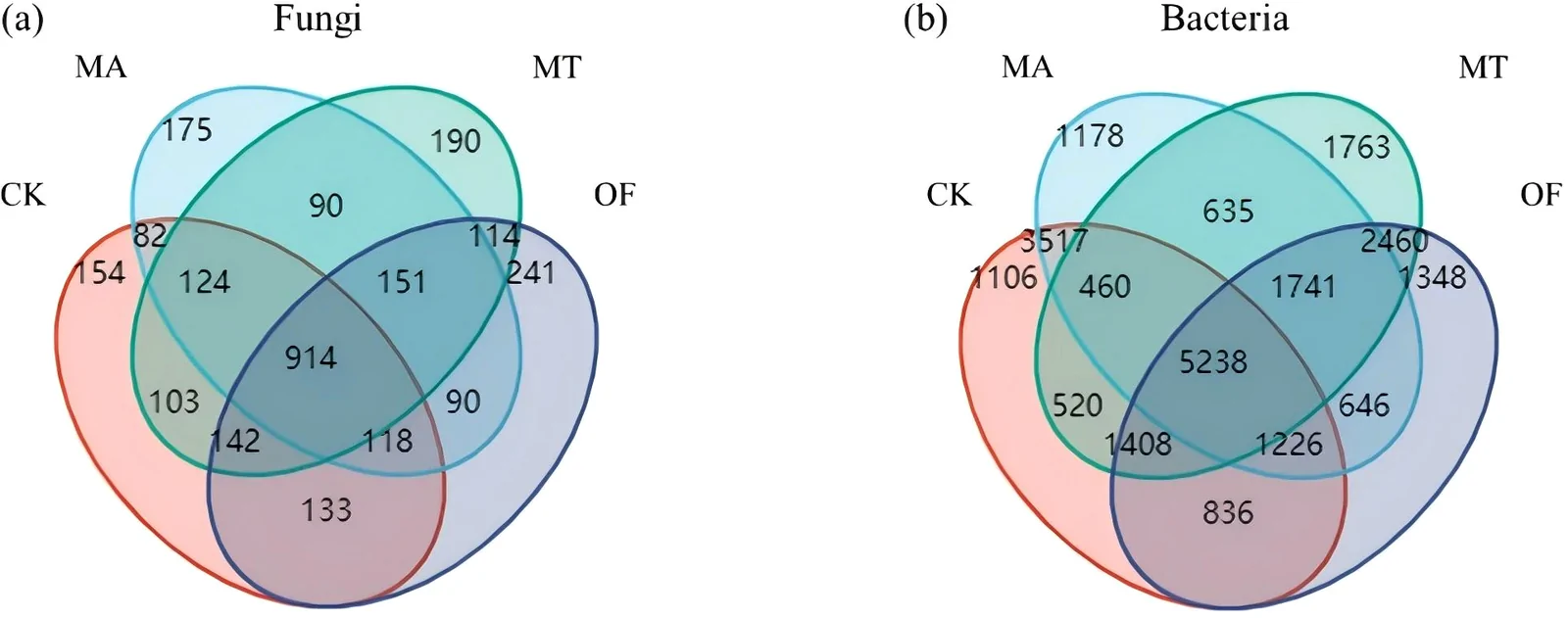
Venn diagram of soil fungi **(a)** and bacteria **(b)**. CK, control check; MA, microbial agent; MT, mowing; OF, organic fertilizer.

As shown in **Table 4**, significant differences (*P<* 0.05) were observed in the partial indices of alpha diversity of soil fungal and bacterial communities under MA, MT, and OF treatments. The MA and MT treatments had no significant effect on the fungal communities compared to the CK, but the Simpson and Shannon indices of the fungal communities were significantly higher (*P<* 0.05) under the OF treatment: 7.78% and 16.37%, respectively (**Table 4**). For bacterial communities, the feature index was significantly higher under the OF treatment than under the MA treatment. This indicates a higher number of microbial species; higher values indicate greater species richness within the sample site. The Simpson and Shannon indices for bacterial communities were significantly higher under the MT and OF treatments than under the MA treatment by 5.32% and 27.53%, respectively. Bacterial cover increased significantly under the MT treatment compared to the control (*P<* 0.05).

**Table 4:** Variance analysis of alpha diversity index of soil bacteria and fungi under different grassland management regimes.

Microbial group	Treat	Feature	ACE	Chao1	Simpson	Shannon	Coverage
Fungi	CK	767.8 ± 172.5a	871.94 ± 230.96a	881.58 ± 235a	0.9 ± 0.09b	5.56 ± 0.99b	99.74 ± 0.14a
	MA	738 ± 69.04a	805.44 ± 84.43a	817.99 ± 76.78a	0.97 ± 0.01ab	6.37 ± 0.4ab	99.8 ± 0.04a
	MT	755.8 ± 58.38a	834.82 ± 49.3a	845.59 ± 46.11a	0.95 ± 0.02ab	6.09 ± 0.39ab	99.8 ± 0.03a
	OF	800 ± 86.7a	881.54 ± 78.56a	884.14 ± 64.24a	0.97 ± 0.01a	6.47 ± 0.48a	99.77 ± 0.08a
Bacteria	CK	5427.6 ± 1368.98ab	8682.06 ± 1706.84a	7820.21 ± 1601.6a	0.97 ± 0.05ab	9.3 ± 2.09ab	96.13 ± 0.89b
	MA	4781.6 ± 1096.19b	8331.78 ± 802.21a	7096.04 ± 620.07a	0.94 ± 0.05b	8.1 ± 2.16b	96.59 ± 0.71ab
	MT	6000.4 ± 1360.02ab	7483.06 ± 2032.25a	7459.32 ± 1911.87a	0.99 ± 0a	10.33 ± 0.32a	97.26 ± 1.03a
	OF	6570.8 ± 1044.17a	8883.44 ± 1478.99a	8680.9 ± 1369.43a	0.99 ± 0a	10.33 ± 0.48a	96.21 ± 0.81ab

Values represent mean ± standard error; Different lowercase letters indicate significant differences at *P* < 0.05; CK, control check; MA, microbial agent; MT, mowing; OF, organic fertilizer.

An analysis using the non-metric multidimensional scaling (NMDS) method based on the Bray-Curtis distance revealed a divergence in the bacterial communities under the different utilization mode treatments (**Figure 6**). The NMDS plots under the different treatments showed that the stress value for the bacteria was less than 0.1, indicating reliability. Furthermore, Permanova and Anosim tests showed that there was no significant difference in the β-diversity of the fungal community under different utilization treatments, whereas the bacterial community was significantly different (*P<* 0.05).

**Figure 6:**
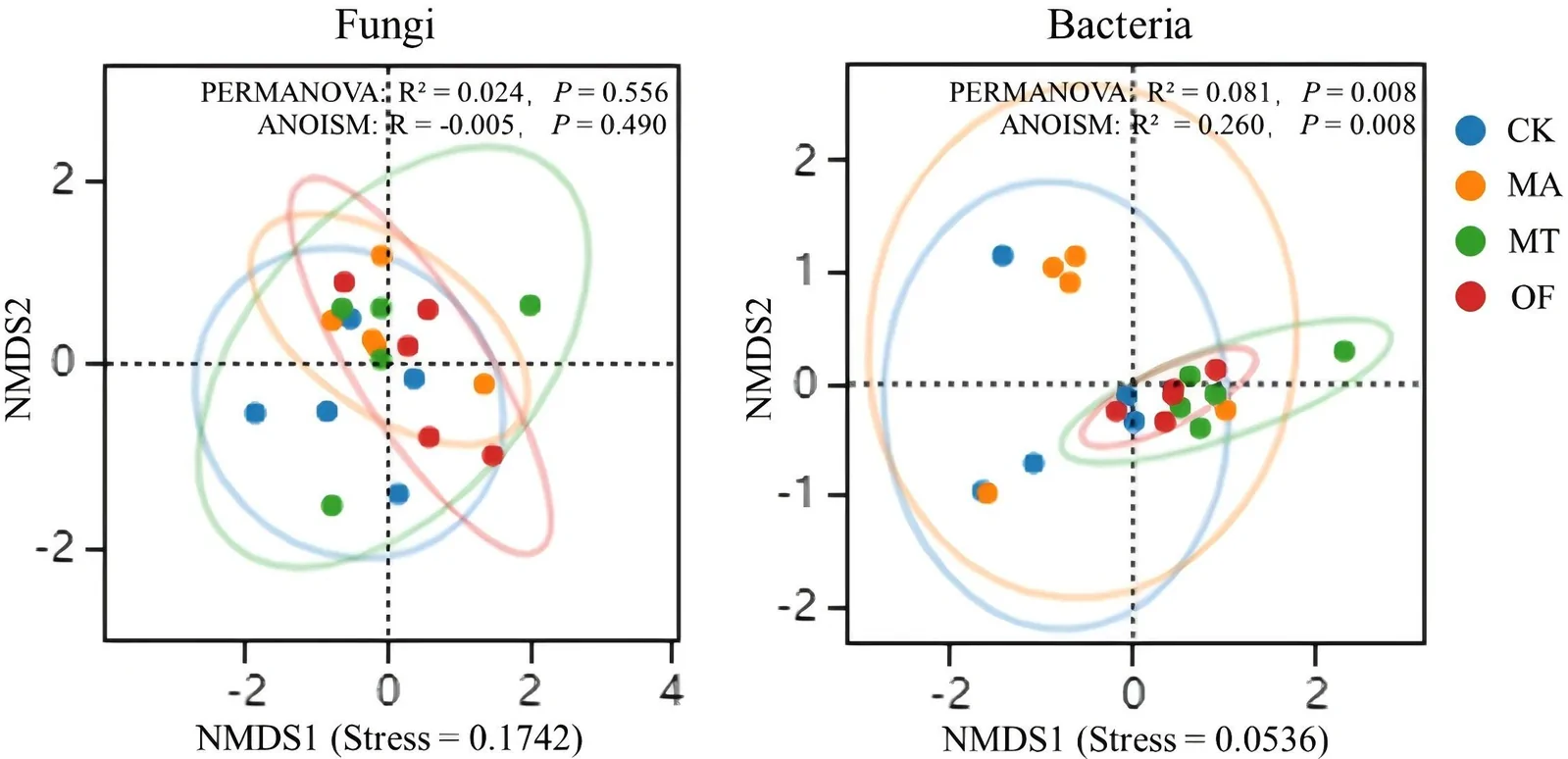
Non-metric multidimensional scaling of soil fungal and bacterial communities. CK, control check; MA, microbial agent; MT, mowing; OF, organic fertilizer.

#### 3.3.3 Effects on the composition of soil microbial communities in grasslands

At the phylum level, the soil fungal community showed the highest relative abundance of Ascomycota in the different treatments, accounting for 69.36%, 54.18%, 59.20%, and 64.50% in CK, MA, MT, and OF, respectively (**Figure 7Aa**). Basidiomycota followed, accounting for 19.98%, 30.74%, 28.15%, and 21.64% in CK, MA, MT, and OF, respectively. The relative abundance of Glomeromycota, Rozellomycota, Chytridiomycota, and Olpidiomycota was less than 1%. At the genus level, *Mortierella* was the dominant genus, accounting for 8.04%, 12.18%, 10.19%, and 11.11% in CK, MA, MT, and OF, respectively. The remaining six fungal genera with a relative abundance greater than 1% (**Figure 7Ab**) were *Cladosporium*, *Clavaria*, *Fusarium*, *Metarhizium*, *Cophophyllus*, and *Exophiala*. Unclassified genera accounted for approximately 26%, and Others accounted for about 40%.

**Figure 7:**
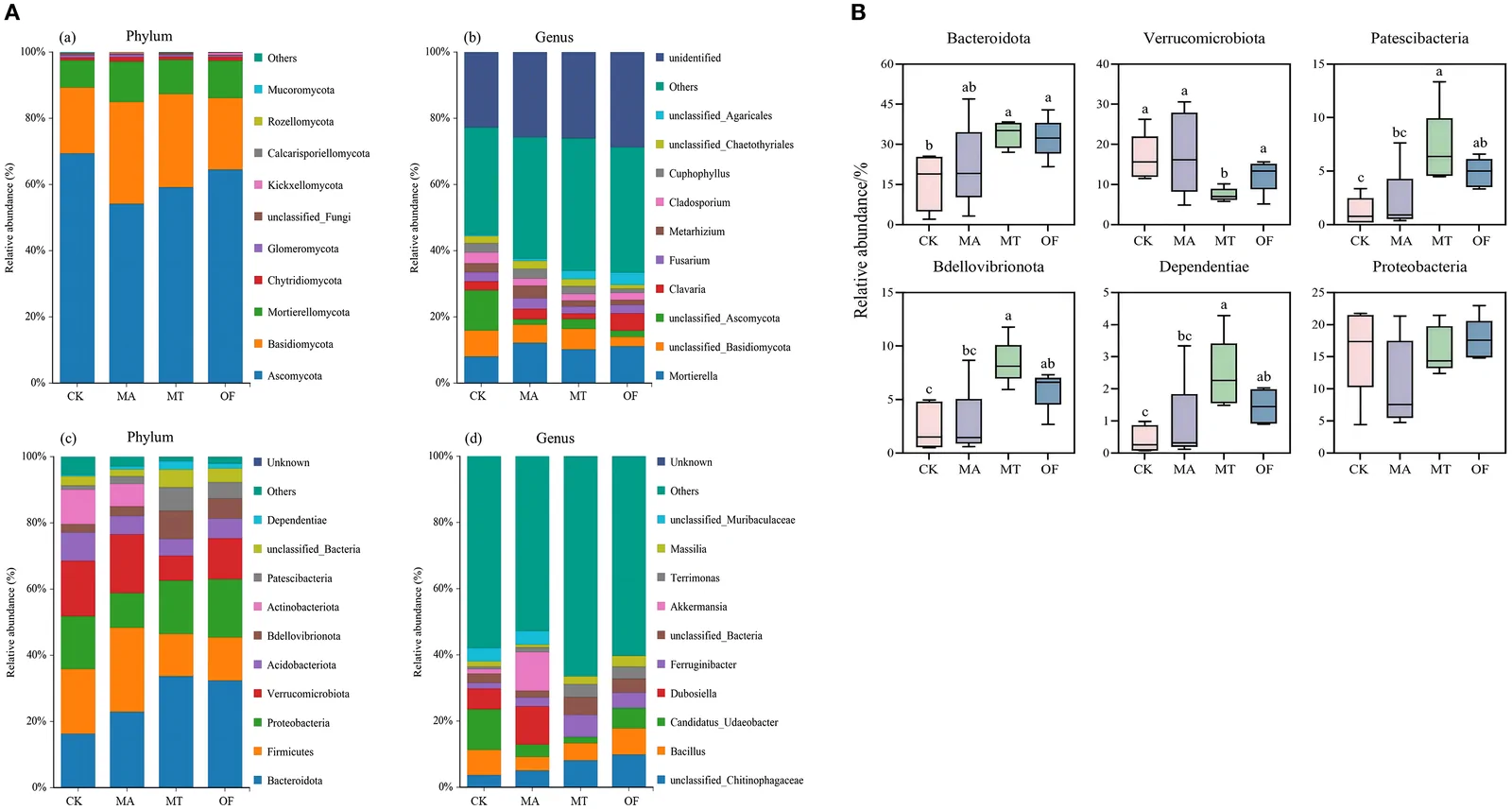
**(A)** Community composition of fungi and bacteria at different taxonomic levels. CK, control check; MA, microbial agent; MT, mowing; OF, organic fertilizer. **(B)** Relative abundance of major bacteria at soil phylum level under different grassland management regimes. Different lowercase letters indicate significant differences at P < 0.05; CK, control check; MA, microbial agent; MT, mowing; OF, organic fertilizer.

At the phylum level, the soil bacterial community was primarily composed of Bacteroidota, which accounted for 16.23%, 22.90%, 33.68%, and 32.36% of the CK, MA, MT, and OF treatments, respectively (**Figure 7Ac**). The relative abundance of Proteobacteria, Verrucomicrobiota, and Firmicutes was greater than 10% under different treatment disturbances. Acidobacteriota, Actinobacteriota, Bdellovibrionota and Patescibacteria all had relative abundances above 1%. At the genus level, five bacterial genera had a relative abundance greater than 1%. Unclassified accounted for approximately 10% of the genera, while Others (relative abundance<1%) accounted for approximately 60% (**Figure 7Ad**).

A MetaStat analysis of the relative abundance of soil bacteria at the phylum level under different utilization modes revealed significant differences among five bacterial taxa: Bacteroidota, Verrucomicrobiota, Patescibacteria, Bdellovibrionota, and the Dependentiae phylum (**Figure 7B**). The MT and OF treatments significantly (*P* < 0.05) increased the relative abundance of the phylum Patescibacteria, Patescibacteria, Bdellovibrionota, and Dependentiae compared to the control, whereas there was no significant difference between the CK and the MA treatments; the MT and OF treatments decreased the relative abundance of the phylum Verrucomicrobiota among the bacteria, and the MT was significantly (*P<* 0.05) lower compared to the control.

High-throughput sequencing combined with FUNGuild function prediction showed the 10 pathogenic fungi with the highest relative abundance at the genus level, among which *Fusarium* had the highest relative abundance of 2.64%. The other species in descending order were *Cladosporium* (2.47%), *Humicola* (1.55%), *Paraphoma* (1.28%), *Alternaria* (1.15%), and *Trichoderma* (1.05%), *Sclerostagonospora* (0.65%), *Knufia* (0.62%), *Chaetocapnodium* (0.30%) and *Ilyonectria* (0.25%) (**Figure 8**).

**Figure 8:**
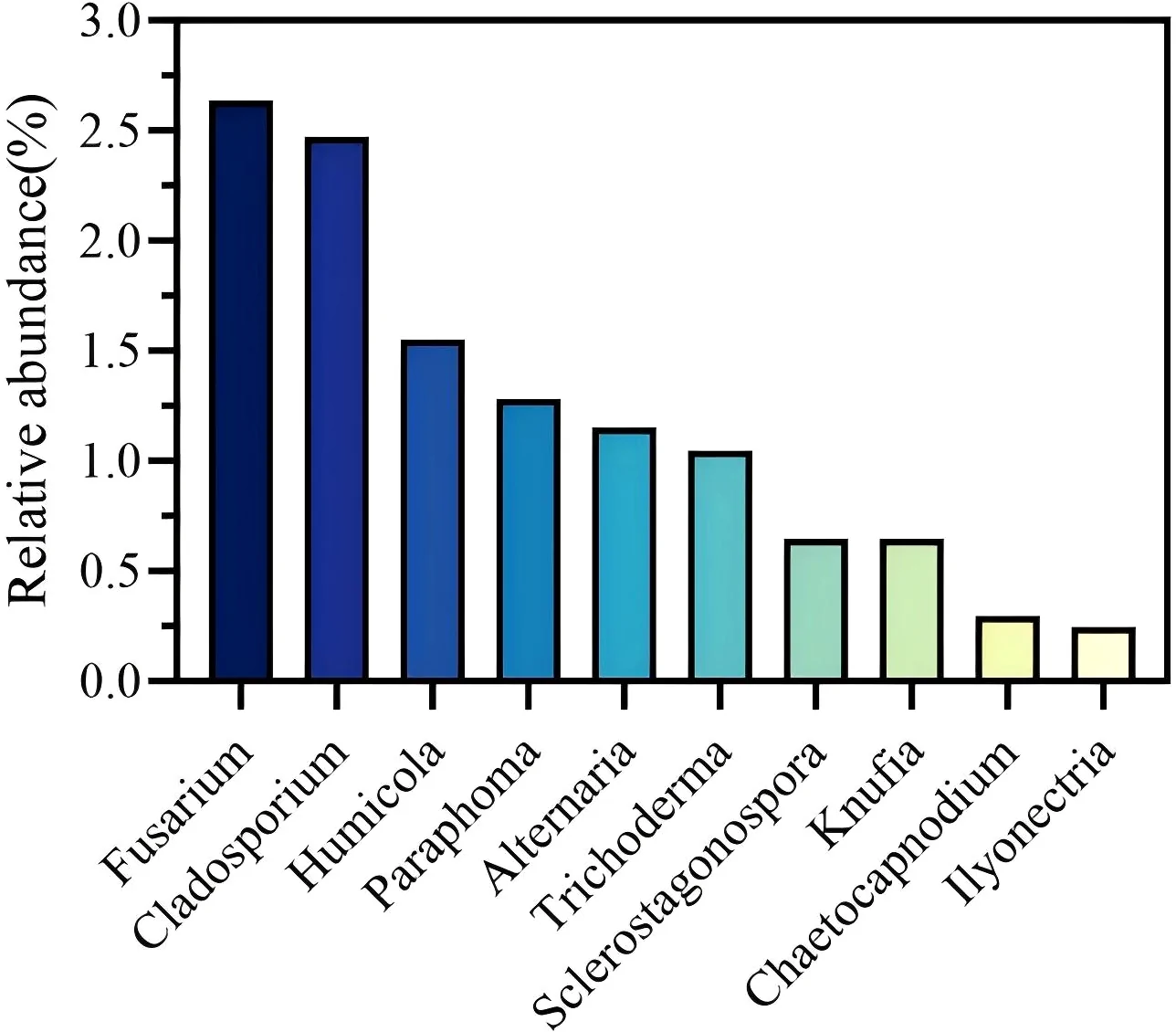
The 10 pathogenic fungi with the highest relative abundance at the genus level.

### 3.4 Correlation analysis of soil microbial community composition and soil physical and chemical indicators

At the phylum level of the fungal community (**Figure 9a**), the relative abundance of Kickxellomycota showed a significant positive correlation (*P<* 0.05) with soil AK content. However, the relative abundance of Calcarisporiellomycota showed a significant negative correlation (*P<* 0.001) with AK content. The relative abundance of Mucoromycota was significantly and positively correlated with soil NN content (*P<* 0.05) and significantly and negatively correlated with SOC content (*P<* 0.05). Moreover, the relative abundance of unclassified_Fungi was significantly and positively correlated with soil TP content (*P<* 0.05) and significantly and negatively correlated with pH value (*P<* 0.05). At the genus level of fungal communities (**Figure 9b**), the relative abundance of *Metarhizium*, *Clavaria*, and *unclassified_Chaetothyriales* were all significantly negatively correlated with soil AK content (*P* < 0.05). The relative abundance of *Cuphophyllus* was significantly negatively correlated with SOC content (*P* < 0.05). At the same time, the relative abundance of *Fusarium* and *Cladosporium* were both significantly negatively correlated with soil NN content (*P* < 0.05).

**Figure 9:**
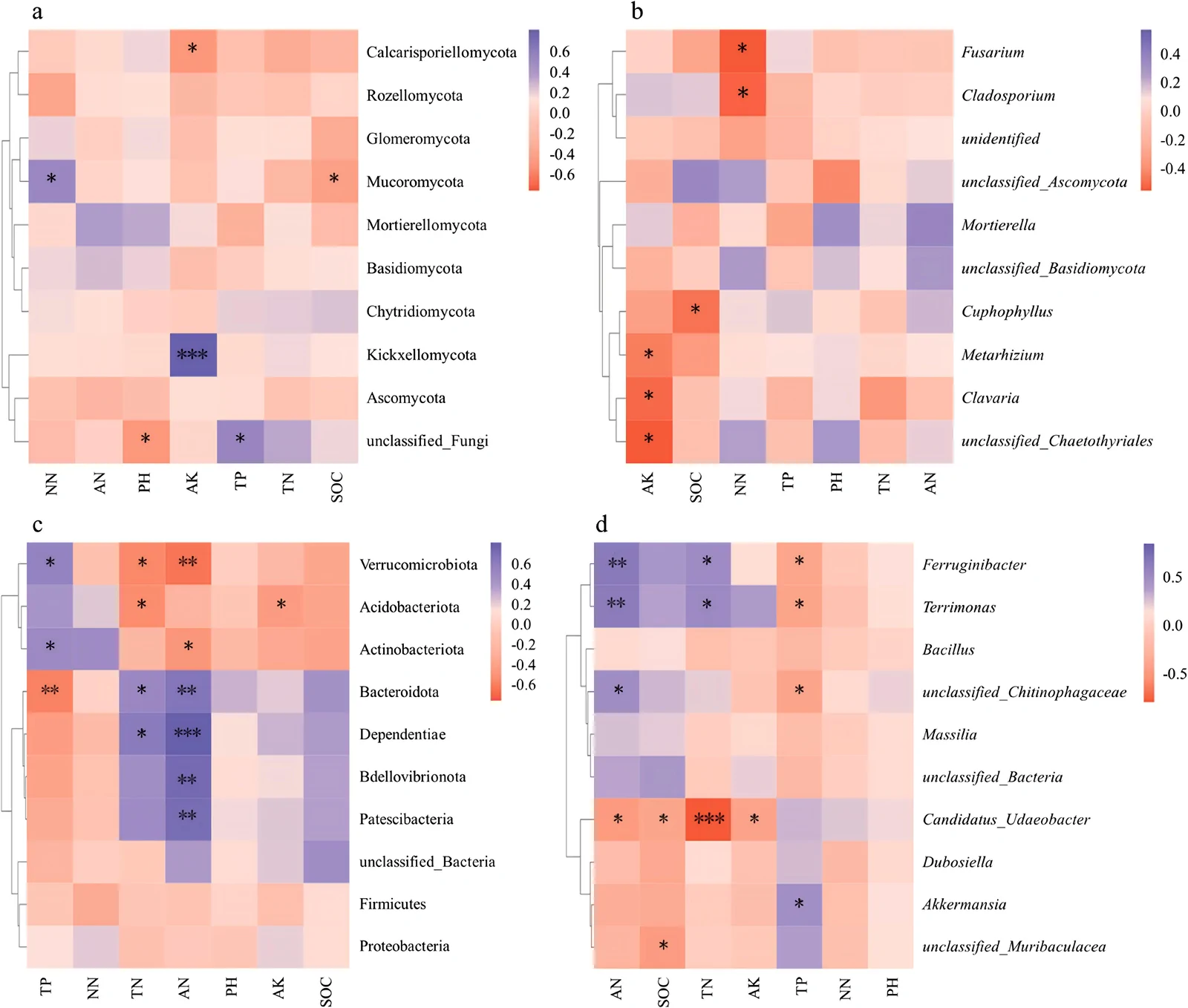
Correlation between soil fungal bacterial community and soil physical and chemical indexes at phylum and genus levels under different treatments. TN, Total nitrogen; TP, Total phosphorus; AN, Ammonium nitrogen; NN, Nitrate nitrogen; AK, Available potassium; SOC, Organic carbon. Purple is positive correlation, red is negative correlation, the darker the color, the higher the correlation; * indicates significant correlation (* is P < 0.05, ** is P < 0.01, *** is P < 0.001).

Meanwhile, at the phylum level of the bacterial community (**Figure 9c**), the relative abundance of Verrucomicrobiota and Actinobacteriota was significantly and positively correlated with soil TP (*P* < 0.05). However, the relative abundance of Bacteroidota was significantly and negatively correlated with soil TP (*P* < 0.01). For soil TN content, the relative abundance of Bacteroidota and Dependentiae was significantly and positively correlated, while Verrucomicrobiota and Acidobacteriota were significantly and negatively correlated (*P* < 0.01). Bacteroidota, Bdellovibrionota, and Patescibacteria (*P* < 0.01) were significantly positively correlated with soil AN content. Verrucomicrobiota (*P* < 0.01) and Actinobacteriota (*P* < 0.05) were significantly negatively correlated with soil AN content. The relative abundance of Acidobacteriota was significantly negatively correlated with soil AK content (*P* < 0.05). At the genus level of the bacterial community (**Figure 9d**), the relative abundance of *Ferruginibacter*, *Terrimonas* (*P* < 0.01), and *unclassified_Chitinophagaceae* (*P* < 0.05) was significantly and positively correlated with soil AN content. *Candidatus Udaeobacter*’s relative abundance was significantly and negatively correlated with soil AN, SOC, soil AK (*P* < 0.05) and soil TN content (*P* < 0.001). For soil TP content, the relative abundance of *Akkermansia* was significantly and positively correlated with it (*P* < 0.05), and the relative abundance of *Ferruginibacter*, *Terrimonas*, and *unclassified_ Chitinophagaceae* were significantly (*P* < 0.05) negatively correlated with soil TP content. For *unclassified_Muribaculacea*, the relative abundance was significantly (*P* < 0.05) negatively correlated with SOC content.

## 4 Discussion

### 4.1 Effects of mowing, microbial agent, and organic fertilizer on plant community structure

Plant community structure and forage production are key indicators for evaluating the ecological and productive effects of grassland management. In this study, microbial agent application significantly increased plant community cover, whereas mowing twice per year significantly reduced community height but markedly increased both fresh and hay forage yields. Organic fertilizer also significantly increased forage yield. These results indicate that the three management measures affected plant communities through different ecological pathways.

The increase in community cover under microbial agent application may be related to the growth-promoting effects of beneficial microorganisms. Beneficial microbial agents can promote plant growth by improving nutrient acquisition, regulating plant physiological processes, enhancing stress resistance, and modifying plant-associated microbial environments (Martínez-Medina et al., 2009; Su et al., 2023). The microbial agent used in this study contained *Bacillus amyloliquefaciens*, *Beauveria bassiana*, and *Trichoderma harzianum*. Among these, Bacillus species are widely reported to participate in nitrogen transformation, phosphorus and potassium mobilization, and plant disease suppression (Cao et al., 2011; Lin et al., 2018). However, microbial agent application also decreased the Pielou and Simpson indices and increased the dominant species presence index, suggesting that this treatment may have strengthened the competitive advantage of certain dominant species rather than uniformly promoting all plant species. Therefore, although microbial agent application improved community cover, its potential effect on community evenness should be considered in long-term grassland management.

The MT treatment significantly reduced plant height, which is a direct consequence of aboveground biomass removal. However, MT treatment also significantly increased the cumulative fresh and hay forage yields (i.e., the sum of biomass harvested at the first mowing in late June and the second mowing in late August). Moderate mowing can reduce shading by tall plants, improve light availability near the soil surface, and stimulate regrowth of remaining tillers and short-statured species (Doležal et al., 2019; Li et al., 2021). In addition, because biomass removed during the early mowing event was included in the annual cumulative yield calculation, the higher yield under MT reflects cumulative forage production during the growing season rather than only standing biomass at the final sampling time. Organic fertilizer increased forage yield mainly by improving soil nutrient availability and supporting plant growth. Previous studies have shown that organic fertilizer can increase plant productivity, soil organic carbon storage, and nutrient availability in grassland ecosystems (Wang et al., 2023; Wei et al., 2025). Compared with MT, OF had a weaker effect on community structure, suggesting that fertilizer application mainly enhanced productivity without strongly reshaping the aboveground community within the short experimental period.

### 4.2 Effects of mowing, microbial agent, and organic fertilizer on plant diseases

Foliar fungal diseases were widely distributed in the Hulunber natural grassland. In this study, visible disease symptoms were recorded on 48 host plant species, and 56 host–disease combinations were identified. Leaf spot was the predominant disease symptom group, occurring on 47 host species, whereas rust and powdery mildew occurred on fewer host species. Disease occurrence also showed clear seasonal dynamics. Rust diseases were more prominent in June, leaf spot diseases became dominant in July and August, and powdery mildew increased toward the late growing season. These results suggest that disease pressure in this grassland community is shaped by both host diversity and seasonal changes in pathogen activity.

At the species level, the responses of disease incidence and severity to different treatments were host-specific. For example, MA and MT reduced the incidence and severity of leaf spot disease in several dominant or common host plants, including *Leymus chinensis*, *Thermopsis lanceolata*, *Pulsatilla turczaninovii*, and *Artemisia tanacetifolia*. These species-level differences may be associated with variation in host susceptibility, plant height, canopy position, and phenology. In species-rich natural grasslands, different host species do not respond uniformly to management measures. Therefore, community-level disease assessment is necessary for evaluating the overall ecological effect of grassland management.

At the functional-group level, MA and MT reduced pathogen loads in major plant functional groups, whereas OF had no significant effect on pathogen load. This pattern indicates that microbial agent application and mowing were more effective than organic fertilizer in reducing visible foliar fungal disease burden. The disease-suppressive effect of microbial agents may be related to direct or indirect inhibition of pathogens, competition for ecological niches, induction of plant resistance, and improvement of plant health (Roberts et al., 2005; Shi et al., 2024). Bacillus and Trichoderma species are widely reported to suppress plant pathogens and promote plant defense responses (Anuar et al., 2023; Zhang et al., 2025). Although *Beauveria bassiana* is mainly known as an entomopathogenic fungus, it may also indirectly influence plant disease dynamics by affecting insect vectors or plant resistance (Harman et al., 2004).

The MT treatment showed a strong disease-reducing effect at the community level. Mowing can remove infected tissues, reduce pathogen-bearing plant material, and improve canopy ventilation and light conditions (Fischer et al., 2012). These changes may lower the humidity and contact frequency required for foliar fungal pathogen infection and spread. However, mowing can also create wounds that may facilitate pathogen invasion, and its effects on disease may vary with mowing time, frequency, host traits, and pathogen type (Kobes et al., 2011). In the present study, mowing twice per year reduced community pathogen load, suggesting that this mowing frequency was sufficient to reduce inoculum accumulation and improve canopy conditions without causing a net increase in disease risk.

In contrast, OF had limited disease-suppressive effects. Organic fertilizer can improve soil fertility and promote plant growth, but its effect on disease is often complex. Appropriately applied organic fertilizer may enhance disease-suppressive soil conditions by increasing microbial activity and improving soil structure, whereas inappropriate nutrient enrichment may favor certain pathogens or increase host tissue density (Ownley et al., 2008). Therefore, organic fertilizer should not be regarded as a direct disease-suppression measure in natural grasslands. Its value may be greater when used as a soil fertility improvement practice in combination with other ecological measures.

### 4.3 Effects of mowing, microbial agent, and organic fertilizer on soil nutrients and soil microbial community

Soil nutrient status is an important environmental factor linking management measures, plant growth, and microbial communities. In this study, MA significantly increased soil total nitrogen, MT increased total nitrogen, ammonium nitrogen, and soil organic carbon, and OF increased soil organic carbon. These results suggest that all three treatments improved soil nutrient status to varying degrees, although the affected nutrient forms differed among treatments.

The increase in soil total nitrogen under MA may be related to the activity of beneficial microorganisms that participate in nutrient transformation and plant growth promotion. Bacillus species can contribute to nitrogen transformation and promote the mobilization of phosphorus and potassium, thereby improving nutrient availability (Cao et al., 2011; Lin et al., 2018). The increases in total nitrogen, ammonium nitrogen, and soil organic carbon under MT may be associated with plant residue retention, root turnover, and changes in plant regrowth after mowing. Mowing can alter litter input, root growth, and soil nutrient cycling, thereby influencing soil carbon and nitrogen pools (Bonanomi et al., 2009; Hu et al., 2021). The increase in soil organic carbon under OF is consistent with the direct input of organic materials from fermented sheep manure and the stimulation of plant growth and microbial activity (Gilmullina et al., 2020).

Soil microbial communities responded differently to management treatments. Fungal community diversity was significantly increased by OF, whereas MA and MT had no significant effect on fungal alpha diversity. This suggests that the additional organic carbon and nutrients supplied by organic fertilizer provided more resources for fungal growth and niche differentiation. Organic inputs can provide energy sources and substrates for soil microorganisms, thereby promoting microbial proliferation and metabolic activity (Xu et al., 2002; Liu et al., 2021). For bacterial communities, MT and OF increased several diversity indices compared with MA, and bacterial community composition differed significantly among treatments. These results indicate that bacterial communities were more sensitive than fungal communities to short-term management disturbances in this natural grassland.

At the taxonomic level, Ascomycota and Basidiomycota were the dominant fungal phyla, while Bacteroidota, Proteobacteria, Verrucomicrobiota, and Firmicutes were important bacterial groups. Ascomycota are important decomposers of complex organic matter and contribute to carbon, nitrogen, and phosphorus cycling, whereas Basidiomycota are often associated with lignin and plant residue decomposition (Lienhard et al., 2014; Zhao et al., 2021). Bacteroidota can efficiently degrade complex organic matter and participate in soil carbon and nitrogen cycling (Pan et al., 2023). More importantly, different treatments changed the relative abundance of several microbial taxa. MT and OF increased the relative abundance of Bacteroidota and several bacterial groups associated with organic matter decomposition and nutrient cycling, while MT decreased Verrucomicrobiota relative to CK. These shifts indicate that management measures did not merely change microbial diversity but also altered the composition of microbial communities involved in soil carbon and nutrient cycling.

### 4.4 Relationships between soil physicochemical properties and soil microbial community composition

The correlation analysis showed that soil physicochemical properties were closely associated with microbial community composition. Soil pH, nitrogen forms, soil organic carbon, and available nutrients are widely recognized as important environmental drivers of soil microbial community structure (Wang et al., 2014). For fungal communities, the relative abundance of *Fusarium* and *Cladosporium* was significantly negatively correlated with soil nitrate nitrogen. This result suggests that soil nitrogen form may be linked to the abundance of certain potential pathogenic fungal genera. Previous studies have shown that nitrate and ammonium nitrogen can influence *Fusarium* growth either directly or indirectly by changing soil chemical conditions (Michel and Mew, 1998). However, because the present study used correlation analysis, these relationships should be interpreted as ecological associations rather than direct causal evidence.

For bacterial communities, Bacteroidota and Dependentiae were positively correlated with soil total nitrogen, while Bacteroidota, Bdellovibrionota, and Patescibacteria were positively correlated with ammonium nitrogen. These bacterial taxa may be involved in organic matter decomposition and nitrogen cycling. In contrast, Acidobacteriota was negatively correlated with total nitrogen and available potassium, which may reflect the preference of some Acidobacteriota members for relatively nutrient-poor soil environments (Joergensen and Emmerling, 2006). At the genus level, *Ferruginibacter*, *Terrimonas*, and *unclassified Chitinophagaceae* were positively correlated with ammonium nitrogen, whereas *Candidatus Udaeobacter* was negatively correlated with several soil nutrient indicators. These results further indicate that soil nutrient availability is an important factor structuring bacterial communities in the Hulunber natural grassland.

## 5 Conclusions and perspectives

### 5.1 Conclusions

This study evaluated the effects of mowing twice per year, microbial agent application, and organic fertilizer application on plant communities, foliar fungal diseases, soil nutrients, and soil microbial communities in the Hulunber natural grassland. The main conclusions are as follows.

First, different management measures affected plant community characteristics and forage production in different ways. Microbial agent application significantly increased plant community cover but slightly reduced community evenness and increased the dominant species presence index. Mowing twice per year significantly reduced community height but markedly increased cumulative fresh and hay forage yields. Organic fertilizer application also significantly increased forage yield, indicating that both MT and OF improved forage production under the conditions of this study.

Second, the three management measures changed soil nutrient status and soil microbial communities. MA increased soil total nitrogen, MT increased total nitrogen, ammonium nitrogen, and soil organic carbon, and OF increased soil organic carbon. OF significantly enhanced fungal alpha diversity, while MT and OF had stronger effects on bacterial diversity and community composition. These results indicate that soil bacterial communities were more sensitive than fungal communities to short-term management disturbances.

Third, soil nutrients were significantly associated with microbial community composition. Several fungal and bacterial taxa showed close correlations with soil nitrogen, available potassium, soil organic carbon, and pH. The negative correlations between *Fusarium* and *Cladosporium* and nitrate nitrogen suggest that soil nutrient status may be related to the abundance of some potential pathogenic fungal genera. However, these relationships represent ecological associations rather than direct causal evidence for changes in foliar pathogen biomass.

Overall, mowing twice per year and microbial agent application showed greater potential for reducing foliar fungal disease burden, while organic fertilizer mainly improved forage production, soil organic carbon, and microbial diversity. These findings provide an ecological basis for developing sustainable grassland management strategies that balance disease reduction, forage production, and soil microbial conservation.

### 5.2 Perspectives

Future studies should further optimize the timing, frequency, and combined application of ecological management measures. In particular, the combined use of mowing and microbial agents may help reduce aboveground pathogen inoculum while improving plant resistance and community health. Organic fertilizer may be incorporated as a soil fertility maintenance measure, but its dosage and application timing should be carefully evaluated to avoid possible increases in pathogen-favorable conditions.

This study was conducted over two growing seasons, and the treatment period was relatively short. Grassland plant diseases and soil microbial communities are strongly influenced by interannual variation in precipitation, temperature, and plant phenology. Therefore, long-term field experiments are needed to determine whether the observed effects of MT, MA, and OF remain stable under different climatic conditions.

## Data Availability

The raw bacterial and fungal amplicon sequencing data have been deposited in the NCBI Sequence Read Archive under BioProject PRJNA1537770 (BioProject record), comprising 20 BioSamples (SAMN63706919–SAMN63706938) and 40 SRA Runs (SRR40963387–SRR40963426).
